# Lower Gastrointestinal Bleeding in Chronic Hemodialysis Patients

**DOI:** 10.4061/2011/272535

**Published:** 2011-10-05

**Authors:** Fahad Saeed, Nikhil Agrawal, Eugene Greenberg, Jean L. Holley

**Affiliations:** ^1^Department of Nephrology and Hypertension, Dartmouth-Hitchcock Medical Center, Lebanon, NH 03756, USA; ^2^Department of Internal Medicine, Westchester Medical Centre, Westchester, NY, USA; ^3^Department of Gastroenterology, Carle Foundation Hospital, Urbana, IL 61801, USA; ^4^Department of Nephrology and Hypertension, University of Illinois at Urbana-Champaign and Carle Physician Group, Urbana, IL 61801, USA

## Abstract

Gastrointestinal (GI) bleeding is more common in patients with chronic kidney disease and is associated with higher mortality than in the general population. Blood losses in this patient population can be quite severe at times and it is important to differentiate anemia of chronic diseases from anemia due to GI bleeding. We review the literature on common causes of lower gastrointestinal bleeding (LGI) in chronic kidney disease (CKD) and end-stage renal disease (ESRD) patients. We suggest an approach to diagnosis and management of this problem.

## 1. Introduction

Gastrointestinal (GI) bleeding is more common in patients with chronic kidney disease and is associated with higher mortality than in the general population [1]. Anemia is a common feature of patients with chronic kidney disease [2, 3]. It is usually normocytic normochromic due to decreased erythropoietin production and red blood cell survival. However, concomitant iron deficiency anemia can also exist due to blood losses during hemodialysis, use of erythropoietin-stimulating agents, or GI bleeding. Initial anemia work up of these patients should therefore include red blood cell indices, absolute reticulocyte count, iron studies, peripheral blood smear, work-up for hemolytic anemia, and an evaluation for GI sources of blood losses if indicated. A clue to the need for GI evaluation for blood loss is in patients who are not replenishing their iron stores despite adequate iron replacement or who demonstrate sudden decrease in stable hemoglobin.

 Physiological mechanisms contributing to an increased bleeding tendency in ESRD patients include uremic platelet dysfunction [4, 5], intermittent heparin use in dialysis, use of antiplatelet agents, and anticoagulants [6].

**Figure 1:**
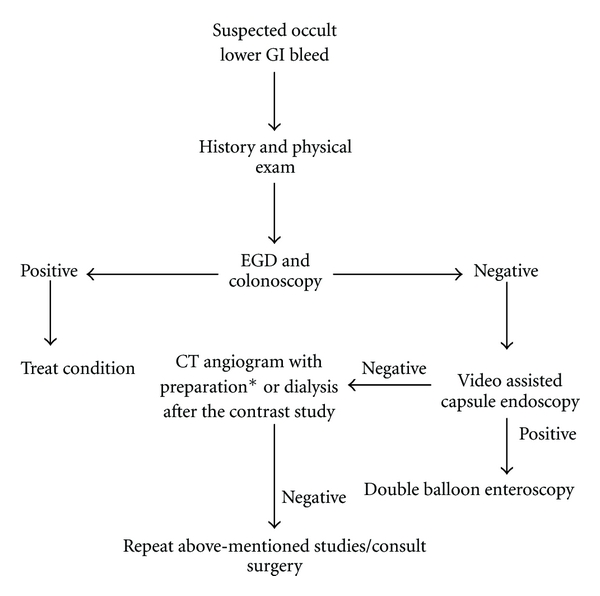
Algorithm to diagnose LGI bleed in CKD patients. *Prepare with normal saline, bicarbonate drip, and acetylcysteine.

**Figure 2:**
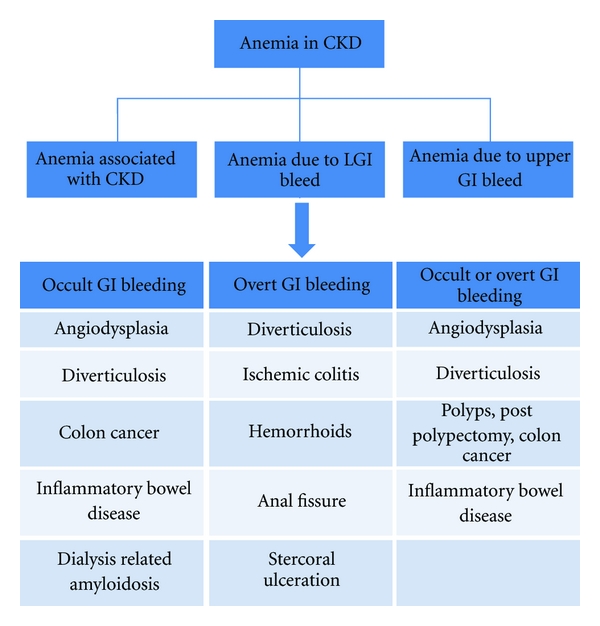
Causes of LGI bleeding in CKD patients.

Anemia itself promotes bleeding diathesis as circulating red blood cells displace platelets toward the vessel wall. This helps maintain their contact with subendothelium at sites of injury. Red blood cells also enhance platelet function by releasing adenosine diphosphate (ADP) and inactivating prostacyclin (PGI) [7]. Thus evaluation of the cause of anemia and its treatment is important for correction of the bleeding diathesis in this patient population. Both upper and lower GI bleeding can contribute to GI losses but this paper will focus on causes of lower GI bleeding in CKD patients.  Figure 1 gives an algorithmic approach to diagnose LGI bleed and Figure 2 highlights the common causes of LGI bleeding in CKD patients.

## 2. Discussion

Lower gastrointestinal tract bleeding is defined as bleeding that occurs distal to the ligament of Treitz. The annual incidence rate of lower gastrointestinal bleeding in the USA ranges from 20.5 to 27 cases per 100,000 adult population at risk (0.03%) [8]. The annual incidence of hospitalization for LGI bleed is estimated to be 20 to 30 per 100,000 persons [9]. In patients who complain of blood in the stool, 10% of cases arise from the upper GI tract segment, proximal to the ligament of Treitz, 5% from the small intestine, and 85% from within the colon [10]. There is lack of specific data regarding the distribution of GI bleeding by location in ESRD patients. Despite the fact that most episodes of lower GI bleeding stop spontaneously without intervention, rebleeding remains a serious problem in 10–40% of patients [9]. Thus, in lower GI tract bleeding, determination of the etiology is important even if the bleeding has stopped. In a study of acute LGI bleed in Canada, the average cost for a patient with LGI bleed was $4,832 Canadian dollars (approximately 3,000 US dollars) with an average length of stay of 7.5 days [11]. Important facts about LGI bleeding in CKD patients are illustrated in Table 1. Each of the common causes of LGI bleeding in CKD patients is reviewed in the following section.

*Angiodysplasia* are vascular ectasias not associated with any familial syndrome, cutaneous, or systemic lesions. They are the most common vascular malformations of the gastrointestinal tract in the general population with a prevalence of 0.82% [12]. Most angiodysplasias occurring in the general population are detected in patients older than 60 years of age [13] although presentation in patients with CKD can be earlier [14]. Angiodysplasias are the leading cause of recurrent LGI bleeding in ESRD patients, accounting for 19–32% of LGI bleeds in those with chronic kidney disease compared with 5-6% of LGI bleeds in the general population [12]. 

Angiodysplasias are most commonly located in the cecum and ascending colon but can be identified in any portion of the GI tract [15]. Histologically, angiodysplasias are small, 5–10 mm, ectatic blood vessels lined by endothelium alone or a thin layer of smooth muscle. The etiology of angiodysplasia is unclear. 

Angiodysplasias tend to be multiple and present with iron deficiency anemia secondary to recurrent GI bleeding. Bleeding secondary to angiodysplasia is more commonly occult and intermittent but massive bleeding can also occur. While bleeding stops spontaneously in about 90% of cases, the tendency to rebleed is seen in 25–47% and can be life threatening in some cases [16]. Factors associated with recurrent bleeding include high bleeding rate, supratherapeutic anticoagulation, and multiple angiodysplastic lesions [16].

The diagnosis of angiodysplasia is mostly accomplished using endoscopic procedures. The typical endoscopically visualized appearance is of a discrete, flat or slightly raised, bright red 5–10 mm fern-like pattern of small dilated veins radiating from a central vessel. Sensitivity of colonoscopy is estimated to be around 81% when the lesion is located in the colon [17]. As angiodysplasia can be located anywhere in the GI tract, visualization of the whole bowel is required. Upper GI endoscopy, push enteroscopy, and wireless capsule enteroscopy are useful in the diagnosis of upper GI angiodysplasias. Selective mesenteric angiography can be utilized in cases of active bleeding. Helical CT angiography is an emerging and promising imaging technique for the noninvasive diagnosis of angiodysplasia and occult GI bleeding. The role of angiography is limited due to the need for intravenous contrast. Its utility in CKD patients applies only to cases of active bleeding who remain undiagnosed despite other investigations. Technicium- (Tc-) labeled scintigraphy is sometimes useful to detect active bleeding; however, it remains of limited use because of the often intermittent nature of bleeding in angiodysplasia and due to its poor sensitivity. 

Angiodysplasias are treated locally with Argon plasma coagulation (APC) [18] or bipolar/heater probe [19]. Angiography may permit localization of a large bleeding lesion with therapeutic embolization or injection of vasopressin [20]. Estrogen, with or without progesterone, has been prescribed in ESRD patients who are not surgical candidates but efficacy of this treatment remains controversial [21–23]. Long-term therapy with octreotide may decrease transfusion requirements and prevent recurrence by decreasing splanchnic blood flow [24]. Angiogenesis inhibitors have also been described as a treatment but evidence for their role is limited [25]. Patients with active bleeding from angiodysplasia who are hemodynamically stable can be managed conservatively with fluid support, and if present, correction of bleeding diathesis and blood transfusions because 90% of these episodes will cease bleeding spontaneously. Iron and erythropoietin replacement needs to be considered. In contrast, hemodynamically unstable patients may require endoscopic obliteration or surgical intervention.

*Diverticulosis* is one of the most common causes of LGI bleeding in ESRD patients. Diverticulosis accounts for approximately 30–50% of cases of lower GI bleeding within the general population [26]. Diverticulosis may not occur with increased frequency in those who are not on dialysis [27]. However, the incidence of LGI bleeding due to diverticulosis in ESRD patients is the same as in the general population. The exception to this is in patients with Adult Polycystic Kidney Disease (APKD) who are on maintenance dialysis and have a higher incidence of diverticular bleeds [23, 28]. In one study, the incidence of diverticulosis in APKD patients on hemodialysis was estimated to be around 83% [28]. In another study, it was around 50% [29]. Incidence is proportional to age with a prevalence of less than 5% at age 40 and 60–65% at the age of 80 years [28]. However, in dialysis patients with kidney disease from any cause, diverticulosis may occur at younger age and may be more severe [30, 31]. 

Diverticuli are outpouchings of intestinal mucosa through the smooth muscle layers and generally occur at the site of penetration of the vasa recta. They are most commonly located in the sigmoid colon and are most commonly false diverticuli covered only by the mucosal and submucosal layers. While the exact cause for the development of diverticulosis is uncertain, intestinal dyskinesias and increased intraluminal pressure in the colon have been postulated. Consumption of a low fiber diet, constipation, and obesity have all been described as risk factors for the development of diverticulosis. Ninety percent of diverticuli are found in the left colon, however, diverticuli from the right colon account for 50% of bleeding [32]. They may cause abdominal pain which may be confused with pain from renal cysts, especially in the setting of APKD. In these patients, the incidence of complications, like colonic perforation, is higher than in the general population and may be increased following renal transplant [28]. The pretransplant detection of diverticulitis is very important, since perforation in the setting of renal transplant carries a mortality rate of 60 percent [33]. This high mortality is due in part to the masking of signs and symptoms of peritonitis [34].

Diverticulosis is a noninflammatory condition and while overt or occult bleeding from diverticula can be associated with symptoms such as nausea and bloating, signs of peritonitis are never consistent with diverticulosis. In cases in which signs of peritonitis are present, diverticulitis should be included in the differential diagnosis but diverticulitis is rarely the cause of GI bleeding [32]. Although diverticulosis has not been proven to be more common in patients on peritoneal dialysis than in the general population, the presence of >10 diverticulae, diverticular size >10 mm, and the presence of diverticula in the ascending, transverse, or descending colon has been associated with an increased risk of peritonitis [35]. Colonoscopy is the initial investigation of choice when diverticulosis is suspected [36]. No significant association has been found between the timing of colonoscopy and diagnostic yield of colonoscopy [37]. Dynamic enhanced helical CT scan can also be used if endoscopy is not diagnostic or is not possible. It is less invasive than angiography and more accurate than nuclear scintigraphy. Contrast-enhanced magnetic resonance angiography (MRA) has been assessed as an investigational tool for detecting bleeding diverticuli. In animal studies, it has demonstrated 100% sensitivity and specificity compared with 78% sensitivity and 72% specificity for nuclear scintigraphy [38]. 

Management of colonic diverticular bleeding includes volume resuscitation. Colonoscopy can be both diagnostic and therapeutic if bleeding diverticula are identified. This is not always possible due to the intermittent nature of bleeding. Arteriography with vasopressin infusion or embolization is usually reserved for patients in whom endoscopy is not feasible or those with persistent or recurrent bleeding and a nondiagnostic colonoscopy [16]. Embolization may carry a 20% risk of infarction [39]. Exploratory laprotomy with partial or total colectomy is considered the definitive diagnostic test when the source of the bleeding diverticuli remains elusive with other techniques.

*Ischemic colitis* arises secondary to a decrease in splanchnic perfusion leading to tissue ischemia as well as reperfusion injury to the bowel wall [40]. Ischemic colitis is more common in ESRD patients due to advanced atherosclerosis and overall compromised circulatory state. In addition, hemodialysis patients have a significantly increased risk for ischemic colitis due to repeated episodes of hypotension and hypovolemia associated with hemodialysis procedures [41]. In one study, the incidence of ischemic colitis in hemodialysis patients was 0.3% per patient-year [42] and in another study ischemic colitis was the most common cause for emergent abdominal surgery, secondary to a nonocclusive vascular emergency [43]. Additional risk factors for nonocclusive mesenteric ischemia in both hemodialysis and peritoneal dialysis patients include aggressive use of recombinant erythropoietin therapy and metastatic calcification [44–46]. A 2009 study reported three cases of nonocclusive mesenteric ischemia in a population of 158 patients on peritoneal dialysis, resulting in an incidence of 1.35% per patient year. As with hemodialysis, the development of acute mesenteric ischemia in patients on peritoneal dialysis may be caused by excessive ultrafiltration [47]. 

Ischemic colitis usually presents as abdominal pain which can be associated with either melena or hematochezia. Abdominal pain may be confused with peritonitis especially in patients on peritoneal dialysis and may result in delay in appropriate treatment and high mortality [44]. Abdominal pain during or after a hemodialysis session particularly when associated with elevated white cell count should raise the suspicion of ischemic colitis [48]. In one small study of patients with mesenteric ischemia, 87% had abdominal pain, fever, and leukocytosis, and 47% had a marked dialysis-associated hypotensive episode prior to the onset of ischemia. In the general population left-sided colonic involvement is more common but in ESRD patients, involvement of the right side of the colon is more common and associated with more severe disease [49, 50]. Findings consistent with an acute abdomen portend a worse prognosis than patients presenting with melena alone [51].

Diagnosis of ischemic colitis is based on the clinical presentation, presence of risk factors, and radiological and endoscopic tests. Colonoscopy is generally needed to establish a definitive diagnosis. Angiography (limited role in CKD patients) and Doppler studies can be employed in cases in which diagnosis is difficult. Treatment of acute colonic ischemia depends upon its severity and the clinical setting. Supportive care including intravenous fluids to maintain colonic perfusion and bowel rest are warranted in almost all cases. Empiric broad spectrum antibiotics can be prescribed in moderate to severe cases. In general, embolectomy, bypass graft, or endarterectomy are rarely used to treat colonic ischemia as large artery obstruction is an extremely uncommon cause of the ischemia. When emergent management is required, a diagnostic laparoscopy can prove to be both diagnostic and therapeutic. 

*Dialysis-related amyloidosis* (DRA) may occur in patients on long-term dialysis. In fact, complications associated with DRA are seen in the majority of patients on dialysis for greater than 20 years [52]. DRA is commonly associated with musculoskeletal complications although gastrointestinal involvement has also been reported. In the cases of gastrointestinal involvement, *β*-2 microglobulin deposits mainly within the muscularis propria of the GI tract wall. This is in contrast to other amyloid proteins which more commonly are deposited within the walls of the arterial system. Reduced motility combined with an increased rigidity secondary to *β*-2 amyloid deposition within the intestinal musculature results in shearing forces and tearing of the mucosa. Coupled with preexisting mucosal ulcerations and coagulopathies, these shear forces can cause significant damage and bleeding within the GI tract [53]. For unknown reasons, involvement of the upper GI tract is more common than the lower GI tract. The most frequent GI manifestation of DRA is GI bleeding with abdominal pain. The severity of bleeding can vary widely. Pseudo-obstruction, perforation, and necrosis are all possible. DRA should be suspected in patients on long-term dialysis without other obvious causes for lower GI bleeding. Endoscopy is nonspecific and can show mucosal folds with or without mucosal ulceration. A diagnosis of DRA must be confirmed by histological documentation of amyloid on biopsy. 

Renal transplantation is the only effective treatment [54]. Biocompatible high-flux hemodialysis membranes may be more effective in removal of the protein but their use does not prevent progression of DRA or the development of new lesions.

*Colon carcinoma and colon polyps* are also important causes of lower GI bleeding in the dialysis population although colon cancer does not appear to be more common in the dialysis population [55]. In the general population, 19% of the cases of lower GI bleeding are attributed to colon cancer and polyps. The risk of colonic polyps in ESRD patients is also not significantly increased as compared to the general population [55]. The specificity of screening for colon cancer among patients with ESRD differs from the general population because dialysis patients have a high incidence of nonmalignant gastrointestinal bleeding abnormalities making guaiac testing misleading. In one study, the incidence of guaiac positive stools was three times higher in asymptomatic dialysis patients compared to non-ESRD controls [56].

*Stercoral ulceration* of the colon is being increasingly recognized as a cause of lower gastrointestinal (GI) tract bleeding in the ESRD population [57]. Pressure induced by hard, large fecal masses induces necrosis and ulceration of colonic mucosa known as stercoral ulcers. There can be single or multiple lesions which can occur throughout the colon but usually occur within the sigmoid colon and rectum [58]. Chronic constipation is the major risk factor for the development of stercoral ulcers. Dialysis patients are more prone to constipation due to phosphate binders such as selevemer [59], fluid restriction, and inactivity as well as slowed bowel motility. Grossly, stercoral ulcers are irregular in shape and sharply delineated from the surrounding colonic mucosa. Microscopically the lesions vary in depth from mucosal ulceration to transmural perforation. Areas with transmural involvement are at the greatest risk of perforation. Chronic ulcers may be complicated by secondary infection, fibrosis, and granulomatous inflammation in response to fecal material.

Stercoral ulcers can present with LGI bleed or features of acute peritonitis. They are associated with a high mortality of around 50% [60]. Diagnosis is made by a history of constipation, demonstration of fecal masses on abdominal imaging along with colonoscopic and histopathological findings. If perforation is suspected, after initial resuscitation and commencement of prophylactic antibiotics, early definitive surgery is warranted. Bleeding ulcers can be successfully treated with endoscopic procedures, including endoscopic multipolar electrocoagulation, Argon plasma coagulation (APC) [61], and injection therapy, [62]. Surgical intervention is indicated in stercoral perforation or failure to control bleeding.

*Inflammatory bowel disease* (IBD) which includes ulcerative colitis and Crohn's disease is another important cause for lower GI bleeding in those with CKD. Although ESRD patients are not at an increased risk of developing IBD, the prevalence of this disease within the general population makes it a significant cause of lower GI bleeding in ESRD patients. Lower GI bleeding is more commonly a presenting symptom in ulcerative colitis. IBD serology should be carefully interpreted in cases of CKD due to the possibility of underlying vasculitis. The utility of the erythrocyte sedimentation rate as an inflammatory marker to predict the disease flare is also limited in this population because an elevated ESR is common, in part due to anemia. Management is similar as in the general population and depends on the severity of disease. 

*Hemorrhoids* are common, affecting approximately 4–10% of the general population and accounting for up 14% of the cases of hematochezia [55]. They are the most common cause of lower GI bleeding in patients under the age of 50 [63]. Hemorrhoids are defined as internal or external depending on their location above or below the dentate line, respectively. The incidence of hemorrhoids is increased within the peritoneal dialysis patient population due to the increased intra-abdominal pressure experienced during peritoneal dialysis [64]. Typically, symptoms increase with the increasing grade of prolapse and most commonly consist of intermittent episodes of painless fresh bleeding that streaks the toilet paper or covers the stools towards the end of defecation [65]. Management of hemorrhoids is based on the clinical presentation and stage of the disease. Adequate fluid and fiber intake is the primary noninvasive treatment of symptomatic hemorrhoids but in ESRD patients more fluid intake can be a problem. Hemorrhoid banding is usually the most effective option. Other options include sclerotherapy, infrared coagulation, Bicap coagulation, and cryotherapy.

*Infectious diarrhea* due to pathogens such as Enterohemorrhagic *E.coli* (EHEC), *Shigella*, *Salmonella*, *Campylobacter* species, and the protozoan *Entamoeba histolytica* can cause visible blood in stools. Generally they can be distinguished from other causes of lower GI bleeding on the basis of clinical setting. Cytomegalovirus (CMV) colitis although uncommon in immunocompetent patients, is important to include in the differential diagnosis of immunosuppressed patients including those with ESRD or postkidney transplantation [66]. ESRD may also increase the risk of acquiring clostridium difficile [67] which may cause bloody diarrhea. Treatment is organism specific based on stool studies.

*Uremic colitis* is now an entity of historical interest only. In the predialysis era, autopsy specimens of untreated uremic patients revealed ulcerations and pseudomembranes, which were termed uremic colitis. Now, due to the widespread availability of hemodialysis, this entity is no longer observed [68].

*Spontaneous colonic perforation* in patients with CKD can occur in association with aluminium-containing antacids, barium studies, and fecal impaction but some percentage of cases remains idiopathic [69]. Traditional causes including diverticulosis should be considered as well. There is a higher risk of colonic perforation during colonoscopy among hemodialysis patients compared with the general population. Beta2-microglobulin deposition is thought to play role in colonic perforation [70]. Colonic perforation has a higher mortality in dialysis patients as compared to the general population [69].

In *summary*, patients with ESRD may develop lower GI bleeding from a variety of sources. Angiodysplasias are the most common cause of lower GI blood loss in this population but other entities need to be considered as well.

##  Conflict of Interests

The authors declare no conflict of interests.

## Figures and Tables

**Table 1:** Important facts about LGI bleed in CKD patients.

(i) Suspect GI blood loss if iron stores are not replenished despite adequate iron replacement or if a sudden drop in stable hemoglobin is seen.	
(ii) Oral iron can cause black stools and give false positive guaiac test in CKD patients.	
(iii) Avoid phosphorus or magnesium based colonic preparations in CKD patients [71].	
